# The impact of baseline shifts towards the heart after image guidance on survival in lung SABR patients

**DOI:** 10.1016/j.radonc.2019.10.018

**Published:** 2020-11

**Authors:** Corinne Johnson-Hart, Gareth Price, Eliana Vasquez Osorio, Corinne Faivre-Finn, Marcel van Herk

**Affiliations:** a Manchester Cancer Research Centre, Division of Cancer Sciences, School of Medical Sciences, Faculty of Biology, Medicine and Health, University of Manchester, Manchester Academic Health Science Centre, UK; b University of Manchester, Manchester Academic Health Science Centre, The Christie NHS Foundation Trust, UK

**Keywords:** NSCLC, SABR, Baseline shifts, IGRT, heart dose

## Abstract

**Background and purpose:**

A recent study of NSCLC patients showed small residual shifts of the high dose region towards/away from the heart after image-guidance were significantly related to overall survival. This study investigates whether the effect is observed in a SABR cohort, who have significantly different baseline outlook and are treated using an imaging protocol matching on the tumour rather than bony-anatomy alone.

**Materials and methods:**

136 NSCLC patients treated with SABR were studied. The mean baseline shift of the tumour in the direction of the heart over the course of treatment was determined for each patient and used to categorise patients into risk groups. Kaplan-Meier survival curves were plotted and multivariable analysis performed to assess significance of the vector shift to the heart alongside common clinical variables.

**Results:**

The vector shift to the heart was independent of all tested clinical variables. A significant difference was seen in patient survival, with patients with shifts towards the heart having significantly worse prognosis as compared to patients with shifts away. Multivariable analysis found a hazard ratio of 1.262 per mm (*p* = 0.013) for the vector shift to the heart, i.e. for every 1 mm shift of the high dose region towards the heart there is a 1.262 higher chance of death.

**Conclusions:**

Baseline shifts towards the heart significantly correlate with overall survival in a cohort of NSCLC SABR patients, with increased risk with increasing shifts towards the heart. These results provide further evidence for the use of stricter heart dose planning constraints for thoracic radiotherapy and suggest a heart planning organ at risk volume may be required for SABR treatments to account for baseline shifts.

## Highlights

• Baseline shifts in the direction of the heart are significantly related to overall survival.
• There is an increased risk with increasing shifts towards the heart.
• Shifts away from the heart reduce the risk, suggesting survival can be improved.
• We recommend stricter heart dose constraints for lung SABR treatment planning.

Radiotherapy plays an important role in the treatment of lung cancer. As the majority of lung cancer patients are elderly with existing comorbidities, surgery is often not a treatment option. Such patients with early stage disease (T1-2N0M0) and peripherally located tumours will be eligible for Stereotactic Ablative Radiotherapy (SABR). SABR is a hypofractionation technique which has been shown to more than double the tumour control rate and improve survival, as compared to conventional radiotherapy in Non-Small Cell Lung Cancer (NSCLC) [1], [2], [3], [4], and as such is regarded as a promising alternative to surgery.

Image-guided radiotherapy (IGRT) has been widely implemented to aid with patient positioning throughout treatment [5], [6]. Deviations in patients’ position from planned, due for example to errors in setting up at the start of each daily treatment fraction, will result in changes in the delivered dose distribution from that prescribed. These differences may affect the probability of both tumour control and normal tissue complications. Many studies have reported superior conformance to the plan when IGRT is introduced [7], [8], [9] and it is this quality assurance that permits the safe delivery of the high doses used in SABR treatments. Direct evidence of the clinical benefits of IGRT, with regard to clinical outcomes, however, is limited.

A recent study by Johnson-Hart et al. [10] looked at the effect of residual setup errors after image-guidance on patient survival. Cohorts of 780 NSCLC patients and 167 oesophagus cancer patients who received radical radiotherapy and mostly offline setup corrections were studied. The residual setup errors after IGRT were estimated from the available Cone-Beam CT (CBCT) image registrations. The group observed no correlations between residual shifts and any clinical variables, yet the small residual setup errors were found to be significantly associated with overall survival. Specifically, residual shifts that move the heart towards the high dose region were found to have a negative effect on survival, as compared to residual shifts that move the heart away from the high dose region. This result remained significant when correcting for common confounding clinical factors. It was concluded that the observed survival difference was related to changes in heart dose - an assumption in line with the results of other studies that found a correlation between heart dose, cardiac events and/or survival [11], [12], [13], [14].

In this study we undertake a similar analysis for a cohort of NSCLC patients treated with SABR, who receive daily imaging with an IGRT protocol that focuses on soft tissue based alignment rather than just bony anatomy. In such patients, baseline shifts of the tumour are known to occur [15] which will cause shifts of the high dose region relative to healthy tissue.

## Methods

136 early stage (overall stage I-II) NSCLC patients treated with stereotactic ablative radiotherapy and for whom CBCT images were accessible for analysis, were retrospectively selected. All applicable information and research governance standards were followed in the preparation of the data (research ethics ref. 17/NW/0060). All patients had peripherally located tumours, lying outside the ‘no-fly zone’, covering 2 cm around the proximal bronchial tree. Patients were treated with one of three fractionation regimes: 54 Gy in 3 fractions, or 60 Gy in either 5 or 8 fractions. All patients were planned using 4DCT scans. The internal Gross Tumour Volume (iGTV) was outlined on the maximum intensity projection image and a uniform 5 mm expansion was applied to obtain the Planning Target Volume (PTV). All patients were verified with daily online CBCT imaging prior to the treatment delivery. A two-stage registration process was used. First CBCT images were registered to the planning scan using a rigid-registration of the bony-anatomy within a region of interest over the thoracic spine (results were not saved). Second, these registrations were modified using a rigid grey-level soft tissue match within the planning iGTV and manually adjusted where deemed clinically necessary to ensure tumour coverage. The translations and rotations from the image registration were applied to the centre of the PTV to derive the appropriate couch shift. If any of the required shifts were greater than the 2 mm action threshold in any direction, then an online correction was performed as follows: from the introduction of SABR at our institution until June 2017, if the couch shifts were greater than the 2 mm action threshold then another CBCT image would be acquired following correction for verification. This process was then iterated (up to 4 times) until coverage was deemed optimal. After June 2017, once sufficient confidence in delivering the treatment technique was obtained, verification images were not acquired after applying couch shifts, unless further imaging was deemed necessary, for example, due to concerns regarding dose to organs at risk.

For each patient and fraction, the planning scan and the last CBCT acquired prior to treatment delivery were selected. This CBCT image was positioned relative to the planning CT including all applied shifts, assuming perfect correction. Then, an individualised bony registration region of interest was generated for each patient on the spine within the region defined by the maximum and minimum superior-inferior extent of the iGTV. A bony-anatomy rigid registration was then performed to retrieve the intermediate (i.e. bone only) match position of the two-stage registration process for each CBCT. The bony-anatomy was used as a surrogate for the heart location (therefore assuming a static heart position) as the heart was often not within the field of view of the CBCTs.

To determine the shift of the high dose region towards/away from the heart, the vector length between the planning target volume (PTV) centre of mass (CoM) – taken to be the position of the high dose region – and the heart CoM was calculated from the planning scan (assuming the CoM to be a representative point for the structure position). The baseline shifts were then applied to the heart position, and this vector length was re-calculated. The difference between these two values determines the shift of the heart towards or away from the high dose region as compared to the plan. The average shift to the heart over all fractions was calculated to obtain the systematic shift.

Univariable analysis of any relationship between clinical variables and the vector shift to the heart was undertaken via Pearson correlation and Analysis of Variance. Tested clinical variables included: age, gender, ECOG performance status (ECOG-PS), overall stage, t-stage, the natural logarithm of the Gross Tumour Volume (GTV, estimated from the motion-adapted GTV, using the method described by Johnson et al. [16]), fractionation scheme, the time between planning and treatment and ACE-27 scale comorbidities. The resulting p-values were adjusted using the Benjamini and Hochberg False Discovery Rate (FDR) method [17], to correct for the effects of multiple comparisons. Elastic net penalised Cox regression with equal ridge regression and LASSO penalty terms was then used to investigate which variables most strongly related to patients’ overall survival.

The median value of the mean vector shift to the heart was used to categorise patients into two groups and Kaplan-Meier survival curves for each group were plotted. Multivariable analysis of both the categorised risk groups and the continuous vector shift magnitudes was performed using Cox regression alongside clinical variables: age, ECOG-PS, the fractionation regime used, the natural logarithm of the Gross Tumour Volume (GTV), the overall comorbidity score and a cardiac specific comorbidity score. The cardiac comorbidity score was determined by combining only cardiac related comorbidities (arrhythmia, coronary artery disease, myocardial infarction and cardiac failure), utilising the same methodology as for ACE-27 comorbidity scoring.

## Results

The mean follow up time for the 136 SABR patients was 456 days. The median value of the vector shift to the heart was −0.59 mm, with a range of −8.99 mm to 8.61 mm.

**Fig. 1:**
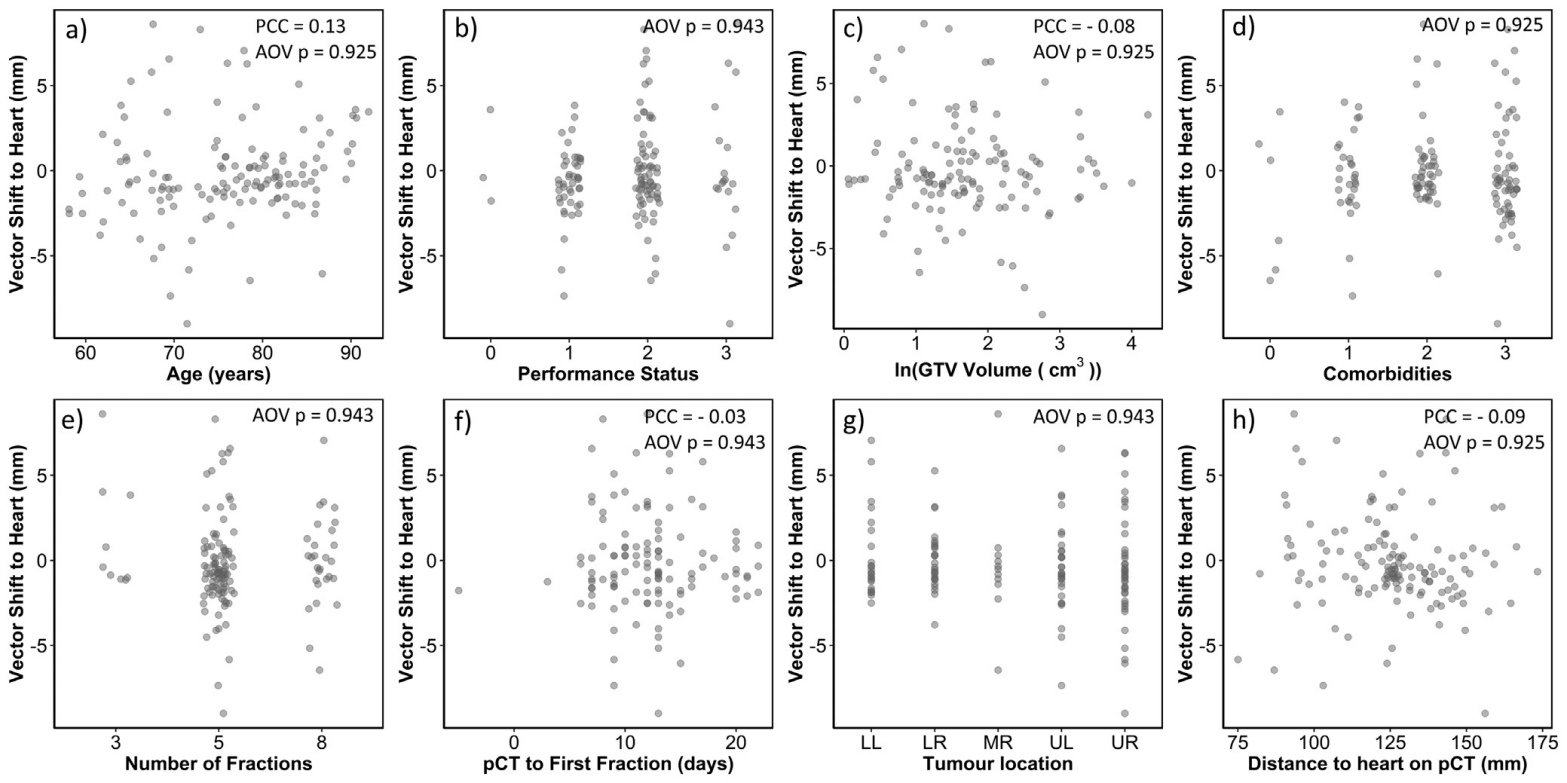
Plots showing the correlation of the vector shift to the heart with (a) patient age, (b) performance status, (c) the natural logarithm of the GTV volume, (d) comorbidities, (e) the number of fractions, (f) the number of days between the planning CT (pCT) and the first treatment fraction, (g) the tumour location (split into lower left (LL), lower right (LR), middle right (MR), upper left (UL) and upper right (UR)) and h) the original distance between the tumour and heart on the planning scan. PCC = Pearson’s Correlation Coefficient; AOV = Analysis of Variance.

Univariable analysis found the vector shift to the heart to be independent of all the clinical variables. Representative plots along with their corresponding Analysis of Variance p-values and Pearson correlation coefficient (where relevant) are shown in Fig. 1.

Variable selection found the mean lateral shift, the vector shift to the heart, age, comorbidities and the natural logarithm of the GTV volume to be significantly related to overall survival. This is the same as in the standard radiotherapy cohort of Johnson-Hart et al., with the addition of comorbidities (which was not available for the previous study).

**Fig. 2:**
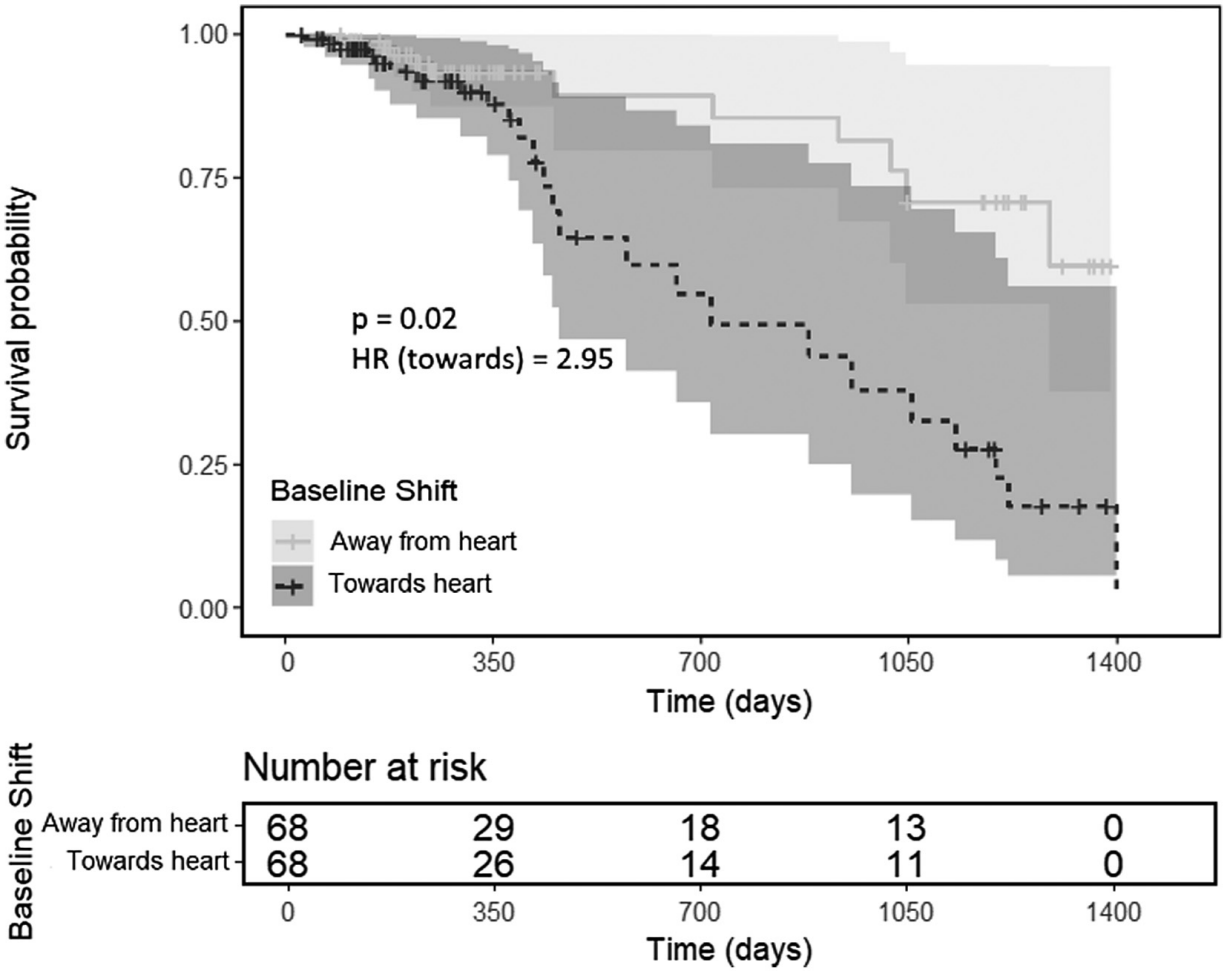
Multivariate Cox regression survival curves for the whole cohort stratified on the median value of the vector shift towards or away from the heart (−0.59 mm), along with the corresponding risk table showing the number of patients at risk at each time point i.e. cases remaining who have not experienced an event or been censored. Patients with high shifts (those >–0.59 mm, meaning that the majority of cases have shifts which move the heart closer to the high dose region) have worse overall survival (*p* = 0.004). The HR gives the hazard of death for patients with shifts towards the heart as compared to patients with shifts away.

When the vector shift to the heart was included as a continuous variable in a multivariable Cox model, correcting for age, ECOG-PS, the natural logarithm of the GTV volume, comorbidity score and cardiac comorbidities, a significant relationship between shifts and survival was seen (Table 1). The detected hazard ratio of 1.262 per mm indicates that with increased shifts towards the heart there is an increased risk of death. This is also demonstrated in Fig. 2, which shows the result of analysing the baseline shift as a binary variable (using the median value of the vector shift to the heart to categorise the patients), where it can be seen that patients with baseline shifts towards the heart have significantly worse overall survival (*p* = 0.004), as compared to those with shifts away.

**Table 1:** Cox regression results.

Variable	*p*-Value	Hazard ratio (95% CI)
Vector shift to the heart (mm)	0.013	1.262 (1.050–1.516)
ECOG-PS (PS0 as reference)		
1	0.370	3.124 (0.259–37.718)
2	0.248	4.229 (0.367–48.731)
3	0.317	3.808 (0.278–52.104)
Age (year)	0.115	1.038 (0.991–1.088)
ln(GTV)	<0.001	2.020 (1.352–3.020)
Fractionation (3# as reference)		
5#	0.471	0.549 (0.107–2.805)
8#	0.501	0.517 (0.076–3.525)
ACE Comorbidity score (0 as reference)		
1	0.143	6.948 (0.520–92.904)
2	0.049	12.889 (1.008–164.780)
3	0.073	12.204 (0.791–188.174)
Cardiac comorbidities (0 as reference)		
1	0.275	0.447 (0.106–1.894)
2	0.703	1.233 (0.422–3.605)
3	0.230	2.136 (0.618–7.380)

Multivariable Cox regression results for the SABR cohort with the vector shift to the heart as a continous variable.

**Fig. 3:**
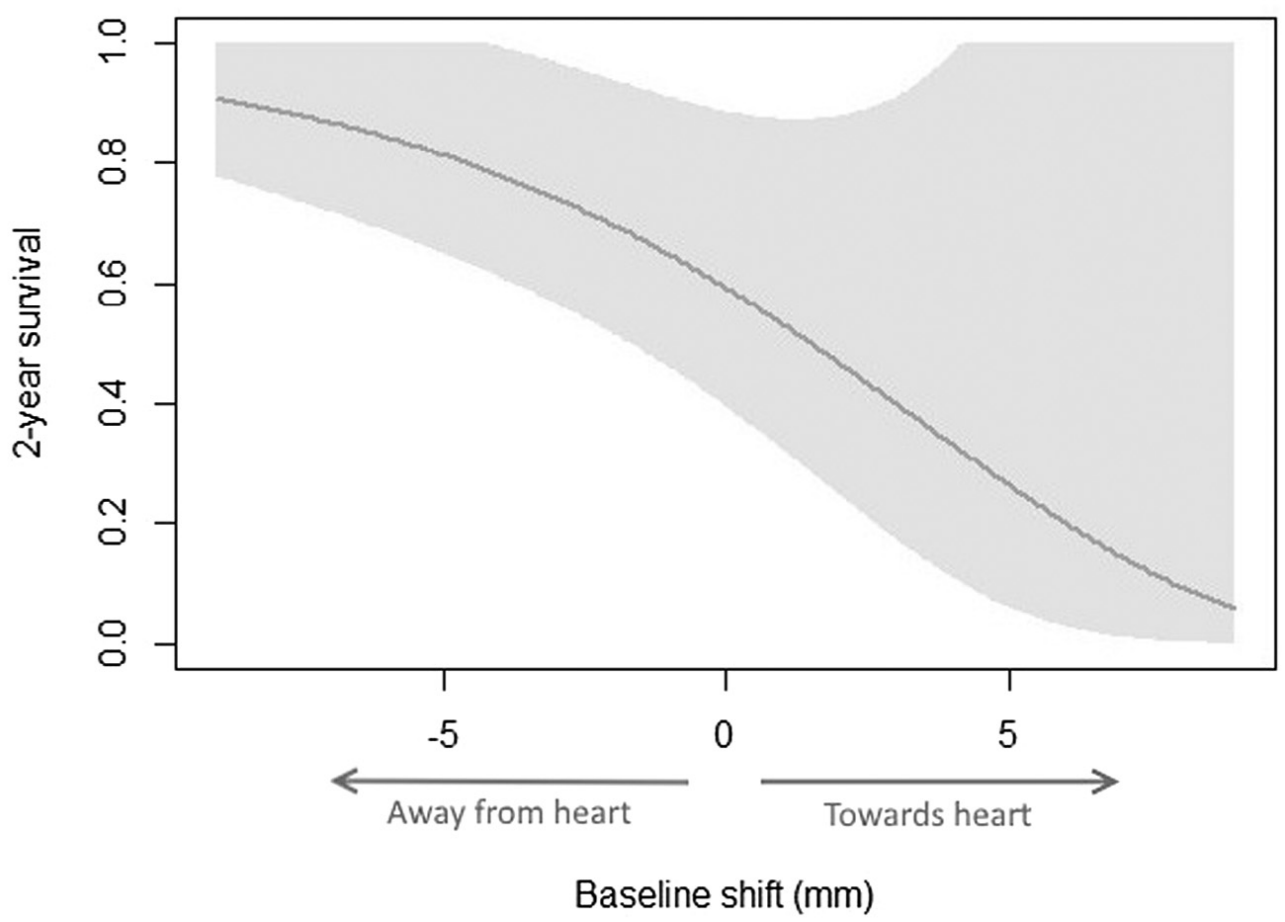
2-year survival probability curve as a function of tumour baseline shifts for a representative patient (as defined by setting the other variables – age, performance status, comorbidity score, cardiac comorbidity score, ln(GTV) and fractionation regime – to the most common values in the cohort). A positive value represents a shift of the high dose region towards the heart. The shaded region represents the 95% confidence interval around this probability curve. This plot shows both the negative effect of shifts towards the heart on 2-year survival probability, as well as the positive effect of shifts away from the heart.

The 2-year survival probability as a function of the baseline shift to the heart for a representative patient is shown in Fig. 3. The representative patient was defined by setting all other variables – age, performance status, comorbidity score, cardiac comorbidity score, ln(GTV) and fractionation regime – to the most commonly occurring value within the cohort. This plot highlights both the negative survival effect of baseline shifts moving the high dose region closer to the heart, but also the positive survival effect of shifts moving the heart away, suggesting treatment improvements are possible.

## Discussion

In this study we explored whether the effects of residual shifts towards and away from the heart on survival, observed in a study by Johnson-Hart et al. [10], are also present in a cohort of lung SABR patients, which employed soft-tissue matching. The main differences in these cohorts are the tumour stage, patient comorbidities and the IGRT imaging protocol. Chi-squared tests showed a significant difference in the distribution of both comorbidities score and stage between the standard and SABR cohorts (*p* < 0.001 in both cases), with SABR patients having greater comorbidities and earlier stage disease. No correlations between the vector shift to the heart and any tested clinical variables were observed (Fig. 1), suggesting that the tumour baseline shifts driving the vector shifts towards and away from the heart are random. These vector shifts are larger in the SABR cohort than those originally observed by Johnson-Hart et al. in a cohort treated with conventional fractionation (with ranges −8.99 mm to 8.61 mm and −4.34 mm to 4.66 mm, respectively). This is mainly a result of the IGRT strategy. In the previous study, bony-anatomy registrations were performed to obtain the appropriate table corrections and the residual setup error was limited by the action threshold for applying these table corrections. For this cohort, soft-tissue matching is performed to derive the required table corrections and thus the shift of the high dose region towards/away from the heart is driven by tumour baseline shifts that are not limited by the imaging protocol.

Analysis of the vector shift to the heart as a continuous variable shows a significant relationship with survival, with increasing shifts towards the heart resulting in an increased risk of death. Similarly, we saw a significant difference in survival between patients with low and high baseline shifts of the tumour when the cohort was split on the median shift value. For a typical patient, the plot of the 2 year survival probability versus the baseline shift (Fig. 3) demonstrates not only the negative effect of baseline shifts towards the heart but the positive effect of shifts away from the heart. Given these shifts are not correlated with clinical variables and the effect is unlikely to be due to lack of tumour coverage, as soft tissue matching is employed, this suggests that the effect we see is related to cardiac dose and associated toxicity. This hypothesis leads to two potential means of improving patient outcome Firstly, stricter cardiac dose constraints may achieve the survival benefit of the high dose region moving away from the heart. Secondly, a planning organ at risk volume (PRV) may be beneficial for counteracting the negative, unavoidable effect of baseline shifts towards the heart. Additionally, it could be beneficial to consider the use of an action threshold which varies depending on the direction of the shift or to review the use of an iGTV in favour of, for example, mid-ventilation planning. However, the effect of these measures would likely also be achieved through the application of a stricter heart dose constraint. A full dosimetric assessment of the effect of these baseline shifts is however required before any firm recommendations can be made. This analysis is currently being performed.

Recent studies using cohorts of conventional radiotherapy lung patients have highlighted the importance of heart toxicity. Stam et al. [18] and McWilliam et al. [19] demonstrated that dose to the heart has a negative impact on patient survival, while Dess et al. [11] showed that pre-existing cardiac disease and heart dose are significantly associated with an increase in cardiac event rates. Fewer studies have reported on cohorts of SABR patients, and as far as the authors are aware, this is the first study to investigate the effect on survival of relative shifts of the tumour and heart in SABR patients. The observed effect is very similar to that reported by Johnson-Hart et al. [10] for patients treated with conventional fractionations, though the hazard ratio is greater in our SABR cohort (1.262 vs 1.091 per mm). This may be due to the mean magnitude of the shifts being greater in the SABR cohort (~2 mm vs ~1 mm for the conventional fractionation cohort). Additionally it could be due to the fact that the majority of patients treated with SABR are medically inoperable and have multiple comorbidities, including pre-existing heart conditions, and thus have poorer overall health as compared to those with locally advanced disease. It has been shown that patients with a history of heart disease are at higher risk of developing cardiac events after SABR [20]. However, the cardiac comorbidity score was not significant on multivariable analysis with overall survival. This could be a result of the multiple comorbidities in this cohort and/or an effect of underlying conditions that have gone undiagnosed and under-reported past medical histories. Additionally, the lack of significance of the cardiac score could be due to the crudeness of the scoring mechanism, which often ranks single and multiple comorbidities in the same way. A more refined method of assessing cardiac comorbidities should be sought and included in future analyses.

As the shifts do not correlate with any clinical variables (Fig. 1), the most likely cause of their observed correlation with survival is the corresponding change in heart dose from that planned. Soft-tissue matching is performed hence the effect cannot be a result of under-dosing of the target. This is in line with the results of previous studies which have demonstrated the detrimental impact heart dose can have on short-term overall patient survival [11], [12], [13], [14]. However the physiopathology of the cardiac damage caused by radiotherapy is not well understood and several studies are ongoing including cardiac imaging aiming to elucidate these mechanisms.

Our study has several limitations. Firstly, the treatment position is obtained by retrospectively applying the imaging action threshold to the recorded shift data, which therefore assumes that the couch correction is always perfect. It would be preferable if patient couch positions were available for each fraction, but this was not the case. Secondly, we assumed a static heart position relative to bone when determining the vector shift to the heart. Several studies have shown this not to be the case, reporting the extent of heart motion due to the beating of the heart throughout the cardiac cycle and respiration [21], [22]. For both of the above limitations, however, it is reasonable to assume that the effect will be distributed evenly between the risk groups, mitigating any bias resulting from our assumptions. However it is likely that the actual hazard ratio is greater as the uncertainty dilutes the effect. Lastly, as well as the primary endpoint of overall survival used in this study, it would be of interest to directly investigate cardiac toxicity. Unfortunately detailed data on post treatment cardiac events was not available for this cohort. Such data is often poorly recorded as patients are not always followed-up in the institution where radiotherapy was delivered. This highlights the importance of routinely following up patients after curative-intent treatment in order to learn about the impact of the treatment on normal tissues. We are currently liaising with public organisations to obtain cause of death data.

In this retrospective study we have shown, for the first time, that there is a significant difference in the survival of SABR patients who have tumour baseline shifts that move the heart closer or further away from the high dose region. In this cohort, tumour based setup corrections are applied, which is possible because there is no mediastinal lymph node involvement. Whilst it would be possible in some circumstances to prioritise corrections such that the heart dose increase is limited, over the GTV error being zero, we feel thatthese results instead provide further evidence for the use of stricter heart dose planning constraints for thoracic radiotherapy and that steep dose gradients on the heart boundary should be avoided where possible e.g. by using a planning organ at risk volume (PRV) around the heart.

## Conflict of Interest.

None.
